# Evaluation of Online Critical Care Fellowship Programs

**DOI:** 10.7759/cureus.35408

**Published:** 2023-02-24

**Authors:** Thai T Donenfeld, Arjun Basnet, Britney M Clemen, Supraja Achuthanandan, Tiffany Lu, Amit Dhaliwal, Nancy Bzadough, Manroop K Gill, Aftab Vadsaria, Jude Tabba

**Affiliations:** 1 Internal Medicine, Maimonides Medical Center, New York, USA; 2 Psychology, William Paterson University, Wayne, USA

**Keywords:** fellowship content, website content, website evaluation, online presence, quality indicator, critical care and hospital medicine

## Abstract

Background

The objective of this study was to assess the accessibility and content of the critical care fellowship websites provided on the Electronic Residency Application Services (ERAS) website.

Methods

Using the online information provided by ERAS, we compiled a list of Accreditation Council for Graduate Medical Education (ACGME)-accredited critical care fellowship programs. Each of the links provided by ERAS was evaluated by a standard search on Google as follows: the program name + “critical care fellowship”. After assembling the working links, those websites were subsequently evaluated based on the program description, application process, and educational content.

Results

We reviewed 59 critical care fellowship programs that were obtained from ERAS. Of the 59 programs, one retracted its participation and was not included in the study, and six other programs were excluded due to repeated links on ERAS, nonworking links, and websites without any content. We analyzed the data collected from the remaining 52 programs. Our data shows a general lack of information being provided to prospective critical care candidates.

Conclusions

ERAS is a major source of information for prospective fellows looking for critical care fellowships in the current match. Unfortunately, the majority of the programs evaluated lack substantial information for prospective candidates. Despite many websites containing adequate information regarding program descriptions, there was a lack of information regarding the application process and educational activities.

## Introduction

The coronavirus disease 2019 (COVID-19) pandemic has had a significant impact on critical care medicine, with approximately 5-20% of patients becoming critically ill and requiring intensive care monitoring [1]. This scenario led to drastic changes in the education and teaching of fellow experience. According to a study by Matta A et al., there was a significant decrease in outpatient pulmonary medicine (bronchoscopies, pulmonary function tests) with a universal increase in inpatient critical care medicine (chest tubes, extracorporeal membrane oxygenation (ECMO)) [1].

The COVID-19 pandemic has made a major impact in the critical care world, significantly changing the resident experience, teaching, and perception of medicine as a whole. According to the Journal of the American Medical Association (JAMA), internal medicine applications show a 6% increase from a 2.7% average, and unsurprisingly, fellowship numbers have also increased [2]. The cause is likely multifactorial; the increased exposure to highly complex diseases and critical patients combined with the decreasing barriers to applying and interviewing due to virtual interviews have drastically changed where applicants will apply. This poses a new challenge to fellowship program directors: to produce a strong online presence where applicants can obtain the necessary information regarding the program in an organized and rapid manner. According to a study by Gaeta T et al., “78% of prospective residents applying reported that information provided in a resident website influenced their decision to apply to a particular program and 41% decided not to apply to at least one program based on the quality of its RWS,” indicating the importance of having a strong online presence [3].

Our culture has adapted to social distancing and virtual interviews so it is critical that these websites have working links, easy access, and sufficient information to provide prospective applicants applying for critical care medicine. There have been multiple studies describing the online content and presence of fellowship programs. A study by Ferre A et al. regarding surgical critical care fellowships revealed that 36 of the 90 programs reviewed (40%) had an inaccurate PD named on the website, and that “only 25% of programs had a website containing a program description, faculty list, curriculum, and current/past fellows list,” showing a surprising lack of information [4]. Another study by Huang B et al. showed that vascular surgery training programs “lack sufficient accessibility, content, organization, design, and user-friendliness to allow applicants to access information that informs them sufficiently” [5].

## Materials and methods

The current list of critical care programs participating in the Match was taken from the Electronic Residency Application Services (ERAS) website (https://services.aamc.org/eras/erasstats/par/display.cfm?NAV_ROW=PAR&SPEC_CD=142). The fellowship programs included in this study participated in the 2023 match. Out of the 59 programs on the ERAS website, one retracted from the Match and six did not have working links to websites for review, bringing the total number of programs to 52. The data to be reviewed was collected through the Google search engine (Bing, Yahoo, etc. were excluded) and through the links provided on ERAS.

Next, a number of parameters were evaluated. Those included: working hyperlinks, Google location, program descriptions, program director, address, phone number, email, application information, special requirements, required rotations, call schedules/call responsibilities, conferences, and any ongoing research. This method is similar to those used in studies of other specialties such as orthopedics, cardiology, and surgical critical care [4,6,7]. The information obtained from the 52 programs was then compared to each other: whether or not links were present, present but not working, not present at all, or repeated. Each hyperlink was then assessed if it could provide a streamlined way to the fellowship website and how many steps it would take to get to the specific fellowship website. As with other studies, a specific Google search was performed for each program: [Program name as seen on ERAS] + “critical care fellowship”, and the location of the link for the program was recorded as its location on Google [4,6,7].

Finally, content gathered from the program websites was evaluated in three categories: program description, the application process, and educational activities. These sections were chosen after reviewing other studies that had used those three larger categories [4,6,7]. The program description included: Google links, a program description + program director, phone numbers, emails, addresses, current + previous fellows, and current employment of alumni. Application information included: the application process, the number of recommendations needed, special application requirements, special deadlines, and benefits/salary. Educational content included: didactics, journal club, rotation/curriculum information, research requirements prior to entering the program, previous program research, any ongoing clinical trials occurring at the program, and clinic/call responsibilities.

## Results

Of the 59 programs provided by ERAS, one program was not participating in the 2023 match and six programs were not evaluated due to a lack of information provided online (i.e non-working websites, no information provided/empty websites, or repeated links from ERAS). Of the total links provided, 52 of the 58 (89.7%) programs were evaluated for program description, educational activities, and application process.

Google links provided by ERAS worked for 84.62% of the programs. Using the standard search approach described above, we found critical care fellowship websites were the first available link on Google 90.3% of the time, with an average position for those websites on Google at 1.09.

Program Description

**Figure 1:**
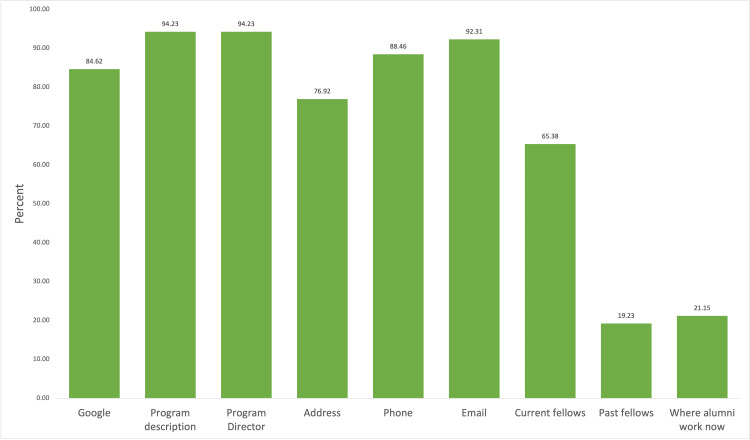
Program Description Program information availability on critical care program websites

The overwhelming majority of programs provided a description of the program (94.23%) and the same number of those programs also included the program director (94.23%). Phones, addresses, and emails to contact the program were not as robust. The email address was most widely given (92.31%), but the address was only given by 40 of the 52 programs (76.92%), and phone numbers were only given by 46 (88.46%). With respect to current and past fellows, we found that programs provided information about their current fellows 65.38% of the time, but only 10 programs had information on past alumni (19.23%) or where their alumni work now (21.15%) (Figure 1).

Educational Activities

**Figure 2:**
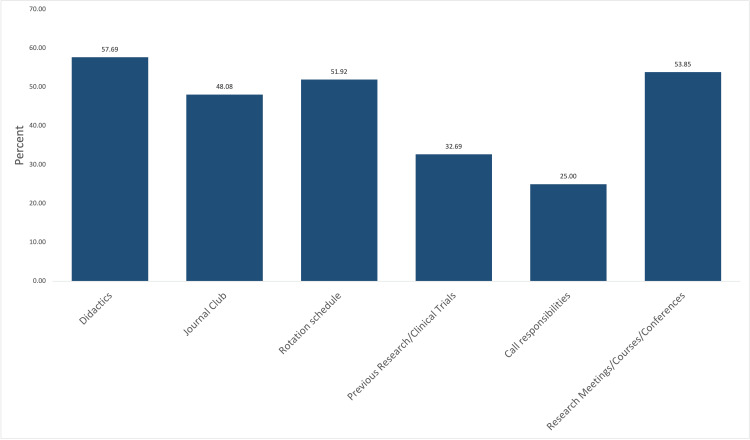
Educational Activities Education activities available on critical care program websites

Evaluation of educational activities was problematic, with only 30 programs providing information on didactics (57.69%). Only 25 programs provided information on journal clubs (48.08%) and 27 programs (51.92%) had information on the rotation schedule. Slightly more than half the programs, 28 total (53.85%), informed prospective fellows about ongoing research or clinical trials. Information about call responsibilities was the lowest in 13 programs (25%) (Figure 2).

Application Process

**Figure 3:**
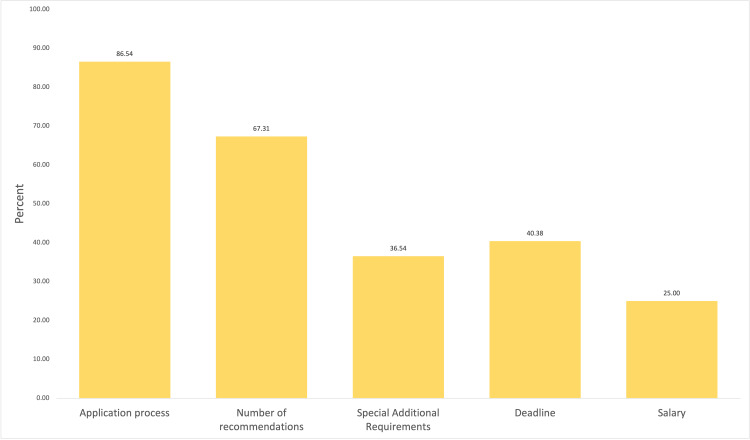
Application Information Application information available on critical care program websites

Of the 52 critical care programs, only 45 (86.54%) had information on their application process, and fewer, 35 (67.31%), programs had information on the number of recommendations required to apply. Nineteen of the 52 programs (36.54%) provided information on any special or additional requirements such as visa or research requirements. The deadline for when applications were reviewed was provided in only 21 programs (40.36%) and information on the salary was available in 13 programs (25%) (Figure 3).

## Discussion

The COVID-19 pandemic has drastically altered the education and structure of medicine. With more residents applying to critical care than before, it has become vital for fellowship programs to have a strong online presence. As previously described, there has been a change in pulmonary critical care education, with a higher emphasis on inpatient critical care due to the pandemic as well as a 6% increase in critical care applications. Virtual tours, online meetings, and group calls have become commonplace since the pandemic due to their safety, convenience, and low cost. It cannot be overemphasized how important it is to have a strong presence, especially as multiple programs have begun switching to online interviews.

Of the 58 programs on ERAS, 52 (89.7%) were evaluated; one program retracted from the match and six programs did not have working websites. Programs were evaluated for program description, educational activities, and application process. Google was the best way to find available programs (84.62%), and the average position of the google link to the program was 1.09. We were pleased to see that the overwhelming majority of programs included both the program director and program description (94.23%). Hospital information, such as an address, phone number, or email, was readily available, but in contrast, current fellows, past fellows, and alumni career information were not provided as readily (65.38%, 19.23%, and 21.15%, respectively).

Similar studies from other specialties, including cardiovascular disease, surgical critical care, and orthopedic trauma surgery, also revealed their program websites' lack of content [4,6,7]. This lack of content can be detrimental to programs attempting to recruit prospective fellows, especially as the number of applicants finding their information online increases. This lack of information appears to be common throughout other specialties when compared to critical care fellowship websites.

Of the 90 surgical critical care fellowship programs listed on the Eastern Association for the Surgery of Trauma (EAST) or American Association for the Surgery of Trauma (AAST) site, 36 programs (40%) had an inaccurate program director in comparison to the 49 (94.23%) websites provided by critical care fellowships and only 25 (20%) surgical critical care fellowship websites contained a program description, faculty list, curriculum, and current/past fellows list [4]. Critical care websites contained less information regarding critical care didactic education (57.69%) when compared to cardiology didactic information (77.6%) [6]. While orthopedic trauma surgery was able to reach 100% with program descriptions, critical care websites came close, at 94.23% [3]. The average overview of the rotation schedule was also lower in critical care as compared to orthopedic trauma surgery and vascular fellowships (51.92% versus 65% and 66%, respectively) [5,7] but was significantly higher than abdominal trauma surgery (17%) [8].

Critical care websites had poor access and provided minimal content regarding the application process and educational activities for prospective fellows. The application process was discussed by 86.54% of websites but only 19 of the 52 websites (67.31%) discussed special requirements for prospective applicants. Special requirements included sponsorships for J1 or H1 visas, which are extremely necessary for international applicants. Application deadlines were only provided by 40.38% of websites. Didactics, journal clubs, and rotation schedules were provided in less than 60% of the programs listed. Research meetings and conferences were provided by 53.85% of programs and only 32.69% of programs included current research/clinical trials.

While our data appear to implicate a lack of general content provided by critical care fellowship websites, which as a whole appear to have less content when compared to other fellowships reviewed in this paper, this paper should not be used as a failing grade. Rather, we hope to shed light on the lack of content available for prospective critical care fellows. There is potential for improvement and better access to fast and reliable information in the application process. Critical care medicine, as a field, is gaining popularity and the number of prospective applicants is growing considerably. Given the internet has become more prevalent in the United States, both among average American and graduate medical students, it has become increasingly important to establish a strong online presence. A survey of 188 emergency medicine residency applicants found that nearly one-half chose not to apply to certain programs because those programs maintained poor websites [3].

We compiled a list of recommendations, based on our analysis, to aid future programs when designing their websites to capture the attention and aid prospective fellows searching for their desired programs. First: This ensures that applicants can contact the program for additional information if necessary. This will ensure the applicants can contact the program for more information should they require it. Second: We recommend having clear tabs with designated pages for information instead of all the information on one page. This can increase page clutter and worsen loading times. Two websites in our study failed to load due to traffic congestion. As stated by Portnet on e-commercial retail "load speed for product category pages has the most impact on sales" [9]. For the loading time of web pages, John Mueller, senior website trends analyst of Google, has recommended aiming for <2-3 secs [10]. While we recognized the difference between commercial sales and fellowship websites the bottom line is simple: longer loading times result in less visibility for the website. Finally, research and research opportunities should be made available to applicants and easily accessible. We found many websites that linked current trials to research being conducted by other departments, which could deter prospective fellows. Including current research the program is participating in will increase the likelihood that highly qualified applicants interested in research applying will rank the program higher.

Our study does have limitations, the most important ones to consider are the subsections we reviewed. While it may be more obvious that applicants would want to see the program director on the fellowship website, it may be less obvious for other parameters. We may not have included some parameters that prospective applicants would want to see as well. The other limitation of the study was the innate risk of searching manually through each website for the information. If the information was well hidden we could mark the content missing.

## Conclusions

Our study was conducted to analyze the content of online information provided by critical care programs. COVID-19 has drastically changed how information is obtained and how important it has become for fellowships to have a strong online presence. Multiple studies in other specialties, including cardiology and surgical critical care, have also shown how important it is to have a strong online presence to recruit prospective fellows.
